# Acetato(aqua){6,6′-dimeth­oxy-2,2′-[ethane-1,2-diylbis(nitrilo­methanylyl­idene)]diphenolato}cobalt(III) methanol disolvate

**DOI:** 10.1107/S1600536812027687

**Published:** 2012-06-23

**Authors:** Gervas Assey, Ray J. Butcher, Yilma Gultneh

**Affiliations:** a Nelson Mandela African Institute of Science and Technology, Department of Materials Science and Engineering, PO Box 447, Arusha, Tanzania; b Department of Chemistry, Howard University, 525 College Street, NW, Washington, DC 2059, USA

## Abstract

In the title complex, [Co(C_18_H_18_N_2_O_4_)(C_2_H_3_O_2_)(H_2_O)]·2CH_3_OH, the Co^III^ atom is hexa­coordinated by water and acetate groups in the axial positions and by the tetra­dentate Schiff base occupying equatorial positions. These axial bonds are longer than the equatorial bonds to the tetra­dentate Schiff base. Two mol­ecules form a dimer through strong hydrogen bonds from the coordinated water of one mol­ecule to the meth­oxy O atoms of an adjoining mol­ecule. There is extensive intra- and inter­molecular O—H⋯O hydrogen bonding between the coordinated water and acetate ligands and the methanol solvent mol­ecules. In addition, there are weak inter­molecular C—H⋯O inter­actions, which link the mol­ecules into a three-dimensional array.

## Related literature

For reports on O_2_ binding of related cobalt complexes, see: Huie *et al.* (1979 ▶); Lindblom *et al.* (1971 ▶). For related dimeric structures formed through hydrogen bonding, see: Huie *et al.* (1979 ▶); Assey *et al.* (2010*b* ▶). For structurally related complexes with included hydrogen-bonded solvent mol­ecules, see: Assey *et al.* (2010*a* ▶,*b* ▶); Ayikoe *et al.* (2010 ▶); Bao *et al.* (2009 ▶); Ayikoé *et al.* (2011 ▶). For the use of cobalt(III)–salen complexes as catalysts, see: Morandi *et al.* (2011 ▶); Haak *et al.* (2010 ▶) and for the potential applications of cobalt–Schiff base complexes for magnetic and/or conducting devices, see: Nabei *et al.* (2009 ▶); Lin *et al.* (2011 ▶).
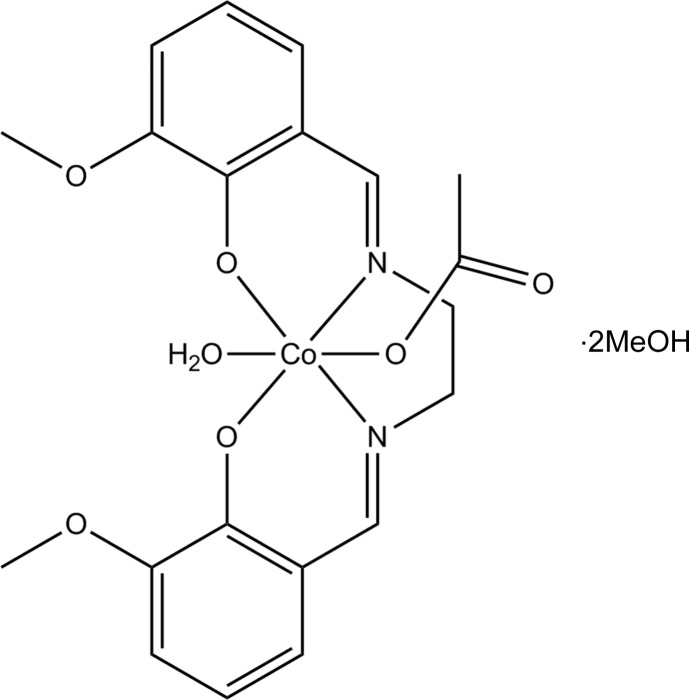


## Experimental

### 

#### Crystal data

[Co(C_18_H_18_N_2_O_4_)(C_2_H_3_O_2_)(H_2_O)]·2CH_4_O
*M* *_r_* = 526.42
Monoclinic, 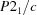

*a* = 9.6306 (3) Å
*b* = 13.4129 (5) Å
*c* = 17.9746 (7) Å
β = 90.716 (3)°
*V* = 2321.67 (15) Å^3^
*Z* = 4
Mo *K*α radiation
μ = 0.80 mm^−1^
*T* = 115 K
0.49 × 0.45 × 0.38 mm

#### Data collection

Oxford Diffraction Xcalibur diffractometer with a Ruby (Gemini Mo) detector
Absorption correction: multi-scan (*CrysAlis RED*; Oxford Diffraction, 2007 ▶) *T*_min_ = 0.916, *T*_max_ = 1.000
16513 measured reflections
7669 independent reflections
5549 reflections with *I* > 2σ(*I*)
*R*_int_ = 0.027

#### Refinement

*R*[*F* ^2^ > 2σ(*F* ^2^)] = 0.040
*wR*(*F* ^2^) = 0.106
*S* = 1.00
7669 reflections
322 parameters
3 restraints
H atoms treated by a mixture of independent and constrained refinement
Δρ_max_ = 0.86 e Å^−3^
Δρ_min_ = −0.45 e Å^−3^

### 

Data collection: *CrysAlis PRO* (Oxford Diffraction, 2007 ▶); cell refinement: *CrysAlis PRO*; data reduction: *CrysAlis PRO*; program(s) used to solve structure: *SHELXS97* (Sheldrick, 2008 ▶); program(s) used to refine structure: *SHELXL97* (Sheldrick, 2008 ▶); molecular graphics: *SHELXTL* (Sheldrick, 2008 ▶); software used to prepare material for publication: *SHELXTL*.

## Supplementary Material

Crystal structure: contains datablock(s) I, global. DOI: 10.1107/S1600536812027687/jj2140sup1.cif

Structure factors: contains datablock(s) I. DOI: 10.1107/S1600536812027687/jj2140Isup2.hkl

Additional supplementary materials:  crystallographic information; 3D view; checkCIF report

## Figures and Tables

**Table 1:** Selected bond lengths (Å)

Co—O2	1.8839 (8)
Co—N1	1.8870 (10)
Co—O1	1.8892 (8)
Co—N2	1.8910 (10)
Co—O11	1.8995 (8)
Co—O1*W*	1.9454 (8)

**Table 2:** Hydrogen-bond geometry (Å, °)

*D*—H⋯*A*	*D*—H	H⋯*A*	*D*⋯*A*	*D*—H⋯*A*
O1*S*—H1*S*⋯O12	0.84	1.83	2.6671 (14)	174
O2*S*—H2*S*⋯O1*S*	0.84	1.95	2.7682 (17)	165
O1*W*—H1*W*1⋯O1^i^	0.80 (1)	1.99 (1)	2.7334 (11)	153 (1)
O1*W*—H1*W*1⋯O3^i^	0.80 (1)	2.32 (1)	2.9124 (13)	131 (1)
O1*W*—H1*W*2⋯O2^i^	0.80 (1)	2.18 (2)	2.8071 (11)	136 (1)
O1*W*—H1*W*2⋯O4^i^	0.80 (1)	2.17 (1)	2.8840 (12)	148 (2)
C9—H9*A*⋯O1*S* ^ii^	0.99	2.52	3.2610 (16)	132
C13—H13*A*⋯O11^iii^	0.95	2.61	3.5380 (15)	165
C12*A*—H12*B*⋯O1	0.98	2.38	3.1807 (15)	139
C8—H8*A*⋯O2*S* ^iv^	0.95	2.55	3.4335 (16)	155
C10—H10*A*⋯O2*S* ^ii^	0.99	2.61	3.5119 (17)	151
C12*A*—H12*C*⋯O2*S* ^v^	0.98	2.62	3.5541 (19)	161

Symmetry codes: (i) 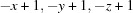; (ii) 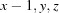; (iii) 
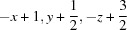
; (iv) 
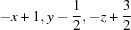
; (v) 
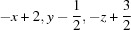
.

**Fig. 1.**
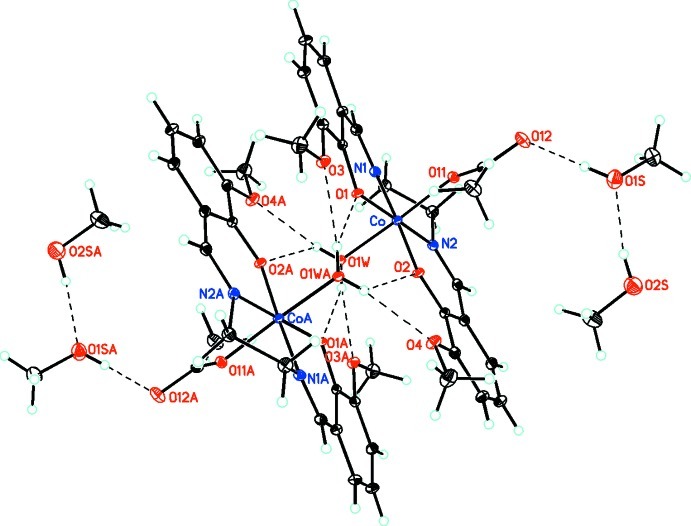
Molecular Structure of the title compound (dimer) along with hydrogen bonding interactions between the coordinated water molecule of one molecule and adjoining oxygen atoms of an adjacent molecule (generated by symmetry codes 1 - x, 1 - y, 1 - z). Hydrogen bonding and weak C—H···O intermolecular interactions are shown by dashed lines.

**Fig. 2.**
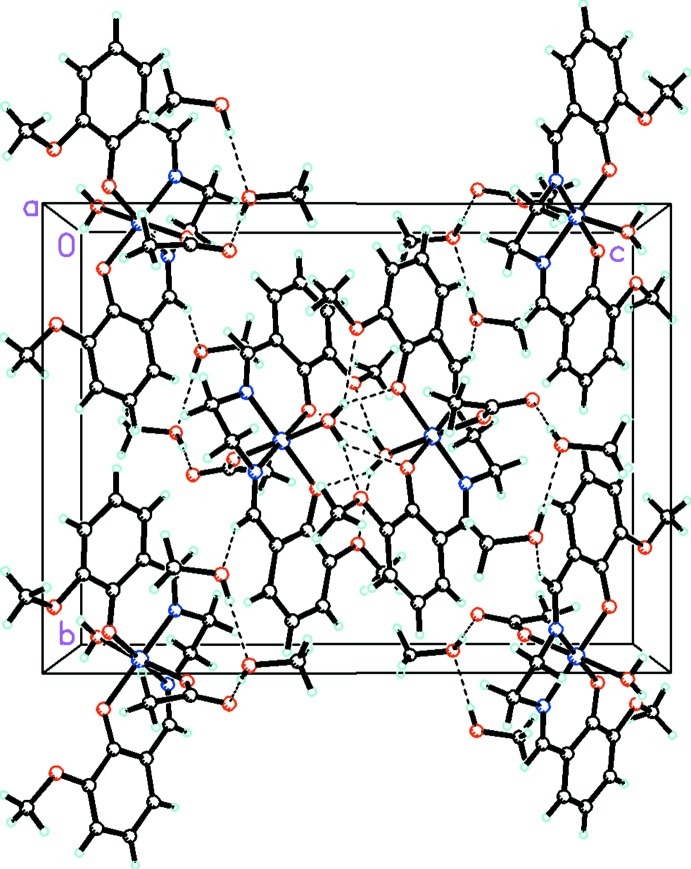
The molecular packing for C22H31CoN2O9 viewed along the a axis. O—H···O hydrogen bonding and weak C—H···O intermolecular interactions are shown by dashed lines.

## supplementary crystallographic information

### Comment

Cobalt Schiff base complexes are of great importance because of their involvement in biological systems. One of their reactions of biological importance is that of binding O_2_ to a metal chelate (Lindblom *et al.* 1971). In recent years, there have been reports about many studies of metal complexes with di-oxygen as one ligand (Huie *et al.*, 1979). The reason behind these studies is to understand the binding between oxygen and transition metals in the proteins that are involved in oxygen transport in living creatures. Another area where the cobalt Schiff bases have find application is that of organic reactions catalysis (Haak *et al.* 2010). Cobalt(III) salen complexes have been described in the literature as catalysts for enantioselective cyclopropagation with diazoacetates in organic media (Morandi *et al.* 2011). Cobalt Schiff base complexes have also been investigated with respect to their potential application for magnetic and/or conducting devices (Nabei *et al.*, 2009; Lin *et al.*, 2011).

In view of the importance of cobalt Schiff base complexes the structure of the title compound, CoC_20_ H_23_N_2_O_7_^.^2(CH_3_OH), has been determined. Schiff base ligands containing a methoxy or ethoxy substituent in the 3 position in the aromatic ring and in a *cis* conformation about the central metal are often involved in interactions where these substituent are either coordinated to a metal (Assey *et al.*, 2010*a*,*b*; Ayikoe, *et al.*, 2010) or form strong hydrogen bonds to a water molecule (or some other suitable solvent such as dimethylformamide) in the cavity created by this conformation (Bao *et al.*, 2009; Ayikoé *et al.*, 2011). In this case, as is found in related Mn and Co complexes (Assey *et al.*, 2010*b*; Huie, *et al.*, 1979), this is achieved by two metal complexes coming together to form a hydrogen bonded dimer. The axially coordinated water molecules of each metal complex form strong hydrogen bonds to the two methoxy groups of the adjoining complex (O1W···O1 2.7335 (11), O1W···O3 2.9124 (13), O1W··· O2 2.8071 (11), O1W···O4 2.8840 (12) Å).

The structure consists of six coordinate Co(III) in a slightly distorted octahedral geometry with both methanol and water occupying the axial positions and a tetradentate Schiff base (N_2_O_2_) which is in the equatorial plane. In addition there are two molecules of solvate methanol in the lattice (Fig. 1). From Table 1 it can be seen that the equatorial metal ligand bond lengths are very similar and vary from 1.8839 (8)Å to 1.8910 (10)Å while the axial bond lengths to the water and acetate moieties are slightly longer at 1.9454 (8)Å and 1.8995 (8)Å respectively. The only slightly distorted nature of the coordination sphere about the Co is emphasized by the fact that the *cis* angles vary from 78.40 (4)° to 94.18 (4)° while the *trans* angles range from 173.73 (3)° to 178.40 (4)°.

There is extensive O—H···O intra- and intermolecular hydrogen bonding between the coordinated water and acetate moieties and the methanol solvate molecules (Fig. 2). In addition there are weak C—H···O intermolecular interactions. These link the structure into a three-dimensional array.

### Experimental

The synthesis of the ligand 3-methoxyethylenediaminebissalicylaldimine was accomplished by the reaction of the solution of (2 g, 33.3 mmol) of ethylenediamine in 10 ml methanol which was added to the solution of *o*-vanillin in 40 ml methanol dropwise using a glass pipette. The mixture was refluxed for 24 h. After solvent evaporation under reduced pressure yellow solids were obtained.

The complex was synthesized by mixing a solution of (0.25 g, 1 mmol) of Co(CH_3_COO)_2_^.^4H_2_O in 5 ml me thanol with a solution of (0.33 g, 1 mmol) 3-methoxyethylenediaminebissalicylaldimine in 3 ml of dichloromethene. The mixture was stirred for 1 h at room temperature, filtered and layered with diethyl ether for crystallization. Crystals suitable for single-crystal X-ray diffraction were obtained by slow evaporation.

### Refinement

H atoms were placed in geometrically idealized positions and constrained to ride on their parent atoms with a C—H distances of 0.95 and 0.99 Å *U*_iso_(H) = 1.2*U*_eq_(C) and 0.98 Å for CH_3_ [*U*_iso_(H) = 1.5*U*_eq_(C)]. The H atoms attached to methanol O were idealized with an O–H distance of 0.84 Å. The water H's were constrained to have a bond length of 0.82 Å and bond angle of 104.5°.

### Crystal data

**Table:**

[Co(C_18_H_18_N_2_O_4_)(C_2_H_3_O_2_)(H_2_O)]·2CH_4_O	*F*(000) = 1104
*M**_r_* = 526.42	*D*_x_ = 1.506 Mg m^−^^3^
Monoclinic, *P*2_1_/*c*	Mo *K*α radiation, λ = 0.71073 Å
Hall symbol: -P 2ybc	Cell parameters from 8159 reflections
*a* = 9.6306 (3) Å	θ = 4.6–32.6°
*b* = 13.4129 (5) Å	µ = 0.80 mm^−^^1^
*c* = 17.9746 (7) Å	*T* = 115 K
β = 90.716 (3)°	Block, black
*V* = 2321.67 (15) Å^3^	0.49 × 0.45 × 0.38 mm
*Z* = 4	

### Data collection

**Table:**

Oxford Diffraction Xcalibur diffractometer with a Ruby (Gemini Mo) detector	7669 independent reflections
Radiation source: Enhance (Mo) X-ray Source	5549 reflections with *I* > 2σ(*I*)
Graphite monochromator	*R*_int_ = 0.027
Detector resolution: 10.5081 pixels mm^-1^	θ_max_ = 32.7°, θ_min_ = 4.6°
ω scans	*h* = −14→10
Absorption correction: multi-scan (*CrysAlis RED*; Oxford Diffraction, 2007)	*k* = −19→19
*T*_min_ = 0.916, *T*_max_ = 1.000	*l* = −26→24
16513 measured reflections	

### Refinement

**Table:**

Refinement on *F*^2^	Primary atom site location: structure-invariant direct methods
Least-squares matrix: full	Secondary atom site location: difference Fourier map
*R*[*F*^2^ > 2σ(*F*^2^)] = 0.040	Hydrogen site location: inferred from neighbouring sites
*wR*(*F*^2^) = 0.106	H atoms treated by a mixture of independent and constrained refinement
*S* = 1.00	*w* = 1/[σ^2^(*F*_o_^2^) + (0.0609*P*)^2^] where *P* = (*F*_o_^2^ + 2*F*_c_^2^)/3
7669 reflections	(Δ/σ)_max_ = 0.003
322 parameters	Δρ_max_ = 0.86 e Å^−^^3^
3 restraints	Δρ_min_ = −0.45 e Å^−^^3^

### Special details

**Table:**

Geometry. All e.s.d.'s (except the e.s.d. in the dihedral angle between two l.s. planes) are estimated using the full covariance matrix. The cell e.s.d.'s are taken into account individually in the estimation of e.s.d.'s in distances, angles and torsion angles; correlations between e.s.d.'s in cell parameters are only used when they are defined by crystal symmetry. An approximate (isotropic) treatment of cell e.s.d.'s is used for estimating e.s.d.'s involving l.s. planes.
Refinement. Refinement of *F*^2^ against ALL reflections. The weighted *R*-factor *wR* and goodness of fit *S* are based on *F*^2^, conventional *R*-factors *R* are based on *F*, with *F* set to zero for negative *F*^2^. The threshold expression of *F*^2^ > σ(*F*^2^) is used only for calculating *R*-factors(gt) *etc*. and is not relevant to the choice of reflections for refinement. *R*-factors based on *F*^2^ are statistically about twice as large as those based on *F*, and *R*- factors based on ALL data will be even larger.

### Fractional atomic coordinates and isotropic or equivalent isotropic displacement parameters (Å^2^)

**Table:**

	*x*	*y*	*z*	*U*_iso_*/*U*_eq_	
Co	0.466441 (15)	0.494930 (12)	0.628307 (8)	0.01070 (3)	
O1	0.51899 (8)	0.38488 (6)	0.56942 (4)	0.01284 (17)	
O2	0.61286 (8)	0.56561 (6)	0.58402 (4)	0.01292 (17)	
O3	0.66160 (8)	0.25427 (7)	0.49906 (5)	0.01703 (19)	
O4	0.81315 (8)	0.63147 (7)	0.50542 (5)	0.0193 (2)	
O11	0.57142 (8)	0.45215 (7)	0.71259 (4)	0.01444 (18)	
O12	0.74146 (9)	0.41453 (8)	0.78855 (5)	0.0254 (2)	
O1S	0.97081 (11)	0.51409 (8)	0.82748 (6)	0.0316 (3)	
H1S	0.9021	0.4805	0.8128	0.038*	
O2S	1.00189 (11)	0.71051 (10)	0.78342 (6)	0.0372 (3)	
H2S	0.9767	0.6530	0.7959	0.045*	
O1W	0.34122 (8)	0.53835 (7)	0.54896 (4)	0.01342 (17)	
H1W1	0.3795 (14)	0.5775 (9)	0.5223 (8)	0.033 (4)*	
H1W2	0.3269 (17)	0.4892 (8)	0.5248 (9)	0.035 (5)*	
N1	0.31519 (9)	0.42594 (8)	0.66977 (5)	0.0138 (2)	
N2	0.40860 (9)	0.60406 (8)	0.68688 (5)	0.0133 (2)	
C1	0.49094 (12)	0.29163 (9)	0.58520 (6)	0.0132 (2)	
C2	0.56595 (12)	0.21670 (9)	0.54734 (6)	0.0149 (2)	
C3	0.73547 (13)	0.18472 (10)	0.45519 (7)	0.0221 (3)	
H3A	0.7886	0.1398	0.4878	0.033*	
H3B	0.7992	0.2205	0.4225	0.033*	
H3C	0.6697	0.1458	0.4249	0.033*	
C4	0.54031 (13)	0.11683 (9)	0.55913 (7)	0.0186 (3)	
H4A	0.5914	0.0680	0.5327	0.022*	
C5	0.43877 (13)	0.08716 (10)	0.61010 (7)	0.0209 (3)	
H5A	0.4220	0.0184	0.6188	0.025*	
C6	0.36441 (12)	0.15766 (10)	0.64706 (7)	0.0192 (3)	
H6A	0.2953	0.1372	0.6811	0.023*	
C7	0.38811 (12)	0.26038 (9)	0.63580 (6)	0.0151 (2)	
C8	0.30021 (12)	0.33125 (10)	0.67197 (6)	0.0162 (3)	
H8A	0.2248	0.3058	0.6998	0.019*	
C9	0.21663 (12)	0.49324 (10)	0.70532 (7)	0.0180 (3)	
H9A	0.1627	0.4573	0.7434	0.022*	
H9B	0.1512	0.5215	0.6680	0.022*	
C10	0.30322 (12)	0.57550 (10)	0.74114 (7)	0.0172 (3)	
H10A	0.2439	0.6334	0.7533	0.021*	
H10B	0.3481	0.5511	0.7875	0.021*	
C11	0.45679 (12)	0.69332 (9)	0.68597 (6)	0.0148 (2)	
H11A	0.4160	0.7408	0.7183	0.018*	
C12	0.56841 (12)	0.72658 (9)	0.63936 (6)	0.0145 (2)	
C13	0.60648 (12)	0.82845 (9)	0.64321 (7)	0.0178 (3)	
H13A	0.5585	0.8722	0.6756	0.021*	
C14	0.71224 (13)	0.86428 (10)	0.60043 (7)	0.0209 (3)	
H14A	0.7367	0.9328	0.6029	0.025*	
C15	0.78416 (13)	0.80023 (10)	0.55325 (7)	0.0197 (3)	
H15A	0.8576	0.8254	0.5239	0.024*	
C16	0.74939 (12)	0.70131 (10)	0.54911 (6)	0.0153 (3)	
C17	0.93798 (13)	0.66034 (12)	0.46918 (8)	0.0283 (3)	
H17A	0.9178	0.7145	0.4341	0.042*	
H17B	0.9758	0.6031	0.4422	0.042*	
H17C	1.0060	0.6832	0.5064	0.042*	
C18	0.63880 (11)	0.66078 (9)	0.59181 (6)	0.0127 (2)	
C11A	0.69919 (12)	0.43003 (10)	0.72351 (7)	0.0168 (3)	
C12A	0.79963 (13)	0.42151 (12)	0.66052 (7)	0.0244 (3)	
H12A	0.8400	0.4871	0.6503	0.037*	
H12B	0.7507	0.3973	0.6160	0.037*	
H12C	0.8737	0.3746	0.6742	0.037*	
C1S	0.96778 (15)	0.52329 (12)	0.90478 (8)	0.0308 (4)	
H1S3	1.0350	0.5740	0.9209	0.046*	
H1S1	0.9917	0.4592	0.9277	0.046*	
H1S2	0.8745	0.5432	0.9200	0.046*	
C2S	0.97663 (17)	0.72400 (13)	0.70700 (9)	0.0356 (4)	
H2S1	0.9926	0.7940	0.6939	0.053*	
H2S2	0.8802	0.7061	0.6951	0.053*	
H2S3	1.0395	0.6814	0.6786	0.053*	

### Atomic displacement parameters (Å^2^)

**Table:**

	*U*^11^	*U*^22^	*U*^33^	*U*^12^	*U*^13^	*U*^23^
Co	0.01352 (6)	0.00968 (7)	0.00896 (6)	−0.00053 (6)	0.00239 (5)	−0.00046 (6)
O1	0.0197 (4)	0.0074 (4)	0.0115 (3)	−0.0009 (3)	0.0038 (3)	0.0000 (3)
O2	0.0161 (3)	0.0090 (4)	0.0138 (4)	−0.0018 (3)	0.0036 (3)	−0.0012 (3)
O3	0.0225 (4)	0.0118 (4)	0.0169 (4)	0.0007 (3)	0.0048 (3)	−0.0034 (3)
O4	0.0208 (4)	0.0178 (4)	0.0195 (4)	−0.0061 (3)	0.0090 (3)	−0.0042 (4)
O11	0.0165 (3)	0.0155 (4)	0.0114 (4)	0.0009 (3)	0.0014 (3)	0.0005 (3)
O12	0.0227 (4)	0.0368 (6)	0.0167 (4)	0.0030 (4)	−0.0017 (3)	0.0068 (4)
O1S	0.0299 (5)	0.0407 (7)	0.0243 (5)	−0.0068 (4)	0.0029 (4)	−0.0023 (5)
O2S	0.0404 (6)	0.0390 (7)	0.0321 (6)	0.0006 (5)	−0.0006 (5)	0.0016 (5)
O1W	0.0178 (4)	0.0106 (4)	0.0118 (4)	−0.0020 (3)	0.0015 (3)	−0.0002 (3)
N1	0.0145 (4)	0.0156 (5)	0.0114 (4)	−0.0009 (4)	0.0023 (3)	0.0013 (4)
N2	0.0159 (4)	0.0134 (5)	0.0107 (4)	0.0016 (4)	0.0018 (3)	−0.0014 (4)
C1	0.0180 (5)	0.0107 (5)	0.0109 (5)	−0.0016 (4)	−0.0023 (4)	−0.0003 (4)
C2	0.0183 (5)	0.0125 (5)	0.0138 (5)	−0.0004 (4)	−0.0019 (4)	0.0004 (5)
C3	0.0238 (6)	0.0196 (6)	0.0231 (6)	0.0053 (5)	0.0041 (5)	−0.0066 (5)
C4	0.0255 (6)	0.0103 (6)	0.0200 (6)	0.0009 (5)	−0.0032 (5)	−0.0017 (5)
C5	0.0288 (6)	0.0103 (6)	0.0236 (6)	−0.0034 (5)	−0.0043 (5)	0.0035 (5)
C6	0.0220 (5)	0.0167 (6)	0.0188 (6)	−0.0055 (5)	−0.0002 (5)	0.0048 (5)
C7	0.0184 (5)	0.0128 (5)	0.0141 (5)	−0.0023 (4)	−0.0003 (4)	0.0005 (5)
C8	0.0168 (5)	0.0196 (6)	0.0122 (5)	−0.0048 (5)	0.0029 (4)	0.0020 (5)
C9	0.0148 (5)	0.0204 (6)	0.0189 (5)	0.0003 (5)	0.0049 (4)	−0.0020 (5)
C10	0.0187 (5)	0.0191 (6)	0.0138 (5)	0.0005 (5)	0.0052 (4)	−0.0030 (5)
C11	0.0168 (5)	0.0153 (6)	0.0124 (5)	0.0025 (4)	0.0000 (4)	−0.0031 (5)
C12	0.0172 (5)	0.0127 (5)	0.0135 (5)	0.0011 (4)	−0.0010 (4)	−0.0019 (4)
C13	0.0238 (5)	0.0117 (5)	0.0177 (5)	0.0014 (5)	−0.0027 (4)	−0.0035 (5)
C14	0.0289 (6)	0.0115 (6)	0.0223 (6)	−0.0042 (5)	−0.0031 (5)	0.0000 (5)
C15	0.0233 (5)	0.0177 (6)	0.0181 (6)	−0.0070 (5)	0.0010 (5)	0.0008 (5)
C16	0.0183 (5)	0.0157 (6)	0.0119 (5)	−0.0016 (4)	0.0007 (4)	−0.0017 (5)
C17	0.0224 (6)	0.0340 (8)	0.0287 (7)	−0.0086 (6)	0.0114 (5)	−0.0055 (6)
C18	0.0153 (5)	0.0110 (5)	0.0118 (5)	−0.0013 (4)	−0.0014 (4)	−0.0006 (4)
C11A	0.0192 (5)	0.0145 (6)	0.0167 (5)	−0.0020 (5)	0.0010 (4)	0.0014 (5)
C12A	0.0183 (5)	0.0338 (8)	0.0211 (6)	0.0070 (5)	0.0044 (5)	0.0040 (6)
C1S	0.0294 (7)	0.0370 (9)	0.0260 (7)	0.0033 (6)	−0.0021 (6)	−0.0010 (7)
C2S	0.0403 (8)	0.0391 (9)	0.0275 (7)	0.0132 (7)	0.0039 (6)	0.0047 (7)

### Geometric parameters (Å, º)

**Table:**

Co—O2	1.8839 (8)	C6—C7	1.4115 (18)
Co—N1	1.8870 (10)	C6—H6A	0.9500
Co—O1	1.8892 (8)	C7—C8	1.4342 (17)
Co—N2	1.8910 (10)	C8—H8A	0.9500
Co—O11	1.8995 (8)	C9—C10	1.5211 (18)
Co—O1W	1.9454 (8)	C9—H9A	0.9900
O1—C1	1.3113 (14)	C9—H9B	0.9900
O2—C18	1.3079 (14)	C10—H10A	0.9900
O3—C2	1.3696 (14)	C10—H10B	0.9900
O3—C3	1.4185 (15)	C11—C12	1.4419 (16)
O4—C16	1.3723 (15)	C11—H11A	0.9500
O4—C17	1.4279 (15)	C12—C18	1.4083 (16)
O11—C11A	1.2786 (14)	C12—C13	1.4162 (17)
O12—C11A	1.2505 (15)	C13—C14	1.3708 (18)
O1S—C1S	1.3956 (18)	C13—H13A	0.9500
O1S—H1S	0.8400	C14—C15	1.3970 (19)
O2S—C2S	1.4038 (18)	C14—H14A	0.9500
O2S—H2S	0.8400	C15—C16	1.3702 (18)
O1W—H1W1	0.803 (11)	C15—H15A	0.9500
O1W—H1W2	0.801 (11)	C16—C18	1.4281 (16)
N1—C8	1.2789 (17)	C17—H17A	0.9800
N1—C9	1.4625 (16)	C17—H17B	0.9800
N2—C11	1.2842 (16)	C17—H17C	0.9800
N2—C10	1.4672 (15)	C11A—C12A	1.5027 (17)
C1—C7	1.4164 (16)	C12A—H12A	0.9800
C1—C2	1.4168 (17)	C12A—H12B	0.9800
C2—C4	1.3790 (17)	C12A—H12C	0.9800
C3—H3A	0.9800	C1S—H1S3	0.9800
C3—H3B	0.9800	C1S—H1S1	0.9800
C3—H3C	0.9800	C1S—H1S2	0.9800
C4—C5	1.4058 (18)	C2S—H2S1	0.9800
C4—H4A	0.9500	C2S—H2S2	0.9800
C5—C6	1.3640 (19)	C2S—H2S3	0.9800
C5—H5A	0.9500		
			
O2—Co—N1	177.81 (4)	N1—C9—H9A	110.5
O2—Co—O1	87.09 (3)	C10—C9—H9A	110.5
N1—Co—O1	92.96 (4)	N1—C9—H9B	110.5
O2—Co—N2	94.18 (4)	C10—C9—H9B	110.5
N1—Co—N2	85.73 (4)	H9A—C9—H9B	108.7
O1—Co—N2	178.40 (4)	N2—C10—C9	106.75 (9)
O2—Co—O11	95.47 (3)	N2—C10—H10A	110.4
N1—Co—O11	86.71 (4)	C9—C10—H10A	110.4
O1—Co—O11	93.85 (4)	N2—C10—H10B	110.4
N2—Co—O11	86.98 (4)	C9—C10—H10B	110.4
O2—Co—O1W	90.00 (3)	H10A—C10—H10B	108.6
N1—Co—O1W	87.81 (4)	N2—C11—C12	124.61 (11)
O1—Co—O1W	89.49 (4)	N2—C11—H11A	117.7
N2—Co—O1W	89.55 (4)	C12—C11—H11A	117.7
O11—Co—O1W	173.73 (3)	C18—C12—C13	120.53 (11)
C1—O1—Co	124.52 (7)	C18—C12—C11	121.78 (11)
C18—O2—Co	126.08 (7)	C13—C12—C11	117.68 (11)
C2—O3—C3	117.14 (10)	C14—C13—C12	120.30 (12)
C16—O4—C17	117.47 (10)	C14—C13—H13A	119.8
C11A—O11—Co	133.96 (8)	C12—C13—H13A	119.8
C1S—O1S—H1S	109.5	C13—C14—C15	120.16 (12)
C2S—O2S—H2S	109.5	C13—C14—H14A	119.9
Co—O1W—H1W1	110.4 (11)	C15—C14—H14A	119.9
Co—O1W—H1W2	104.6 (11)	C16—C15—C14	120.38 (12)
H1W1—O1W—H1W2	107.0 (14)	C16—C15—H15A	119.8
C8—N1—C9	121.69 (10)	C14—C15—H15A	119.8
C8—N1—Co	125.95 (8)	C15—C16—O4	125.54 (11)
C9—N1—Co	112.23 (8)	C15—C16—C18	121.52 (11)
C11—N2—C10	120.33 (10)	O4—C16—C18	112.94 (11)
C11—N2—Co	127.27 (8)	O4—C17—H17A	109.5
C10—N2—Co	112.27 (8)	O4—C17—H17B	109.5
O1—C1—C7	124.67 (11)	H17A—C17—H17B	109.5
O1—C1—C2	117.72 (10)	O4—C17—H17C	109.5
C7—C1—C2	117.59 (11)	H17A—C17—H17C	109.5
O3—C2—C4	125.31 (11)	H17B—C17—H17C	109.5
O3—C2—C1	113.22 (10)	O2—C18—C12	125.72 (10)
C4—C2—C1	121.46 (11)	O2—C18—C16	117.18 (10)
O3—C3—H3A	109.5	C12—C18—C16	117.10 (11)
O3—C3—H3B	109.5	O12—C11A—O11	118.92 (11)
H3A—C3—H3B	109.5	O12—C11A—C12A	119.12 (11)
O3—C3—H3C	109.5	O11—C11A—C12A	121.96 (11)
H3A—C3—H3C	109.5	C11A—C12A—H12A	109.5
H3B—C3—H3C	109.5	C11A—C12A—H12B	109.5
C2—C4—C5	120.17 (12)	H12A—C12A—H12B	109.5
C2—C4—H4A	119.9	C11A—C12A—H12C	109.5
C5—C4—H4A	119.9	H12A—C12A—H12C	109.5
C6—C5—C4	119.66 (12)	H12B—C12A—H12C	109.5
C6—C5—H5A	120.2	O1S—C1S—H1S3	109.5
C4—C5—H5A	120.2	O1S—C1S—H1S1	109.5
C5—C6—C7	121.33 (11)	H1S3—C1S—H1S1	109.5
C5—C6—H6A	119.3	O1S—C1S—H1S2	109.5
C7—C6—H6A	119.3	H1S3—C1S—H1S2	109.5
C6—C7—C1	119.78 (11)	H1S1—C1S—H1S2	109.5
C6—C7—C8	118.98 (11)	O2S—C2S—H2S1	109.5
C1—C7—C8	121.08 (11)	O2S—C2S—H2S2	109.5
N1—C8—C7	125.23 (11)	H2S1—C2S—H2S2	109.5
N1—C8—H8A	117.4	O2S—C2S—H2S3	109.5
C7—C8—H8A	117.4	H2S1—C2S—H2S3	109.5
N1—C9—C10	106.10 (9)	H2S2—C2S—H2S3	109.5
			
O2—Co—O1—C1	156.99 (9)	C5—C6—C7—C1	0.00 (18)
N1—Co—O1—C1	−25.20 (9)	C5—C6—C7—C8	175.51 (11)
O11—Co—O1—C1	61.70 (9)	O1—C1—C7—C6	178.37 (11)
O1W—Co—O1—C1	−112.98 (9)	C2—C1—C7—C6	0.33 (16)
O1—Co—O2—C18	172.53 (9)	O1—C1—C7—C8	2.96 (17)
N2—Co—O2—C18	−6.52 (9)	C2—C1—C7—C8	−175.08 (10)
O11—Co—O2—C18	−93.88 (9)	C9—N1—C8—C7	176.95 (11)
O1W—Co—O2—C18	83.04 (9)	Co—N1—C8—C7	−7.49 (17)
O2—Co—O11—C11A	−31.86 (12)	C6—C7—C8—N1	175.92 (11)
N1—Co—O11—C11A	148.33 (12)	C1—C7—C8—N1	−8.64 (18)
O1—Co—O11—C11A	55.58 (12)	C8—N1—C9—C10	139.65 (11)
N2—Co—O11—C11A	−125.78 (12)	Co—N1—C9—C10	−36.47 (11)
O1—Co—N1—C8	20.37 (10)	C11—N2—C10—C9	150.81 (11)
N2—Co—N1—C8	−160.54 (10)	Co—N2—C10—C9	−33.08 (11)
O11—Co—N1—C8	−73.33 (10)	N1—C9—C10—N2	43.52 (12)
O1W—Co—N1—C8	109.74 (10)	C10—N2—C11—C12	174.50 (10)
O1—Co—N1—C9	−163.71 (8)	Co—N2—C11—C12	−0.97 (17)
N2—Co—N1—C9	15.38 (8)	N2—C11—C12—C18	−2.50 (18)
O11—Co—N1—C9	102.60 (8)	N2—C11—C12—C13	177.98 (11)
O1W—Co—N1—C9	−74.34 (8)	C18—C12—C13—C14	0.19 (18)
O2—Co—N2—C11	4.50 (10)	C11—C12—C13—C14	179.72 (11)
N1—Co—N2—C11	−173.30 (10)	C12—C13—C14—C15	−0.64 (19)
O11—Co—N2—C11	99.77 (10)	C13—C14—C15—C16	0.24 (19)
O1W—Co—N2—C11	−85.47 (10)	C14—C15—C16—O4	−179.43 (11)
O2—Co—N2—C10	−171.28 (7)	C14—C15—C16—C18	0.62 (18)
N1—Co—N2—C10	10.92 (8)	C17—O4—C16—C15	9.33 (17)
O11—Co—N2—C10	−76.01 (8)	C17—O4—C16—C18	−170.72 (10)
O1W—Co—N2—C10	98.75 (8)	Co—O2—C18—C12	5.24 (16)
Co—O1—C1—C7	17.82 (15)	Co—O2—C18—C16	−175.66 (7)
Co—O1—C1—C2	−164.14 (8)	C13—C12—C18—O2	179.73 (11)
C3—O3—C2—C4	3.12 (16)	C11—C12—C18—O2	0.22 (18)
C3—O3—C2—C1	−176.12 (10)	C13—C12—C18—C16	0.62 (16)
O1—C1—C2—O3	1.04 (15)	C11—C12—C18—C16	−178.88 (10)
C7—C1—C2—O3	179.22 (10)	C15—C16—C18—O2	179.78 (11)
O1—C1—C2—C4	−178.23 (10)	O4—C16—C18—O2	−0.17 (14)
C7—C1—C2—C4	−0.05 (16)	C15—C16—C18—C12	−1.04 (17)
O3—C2—C4—C5	−179.75 (11)	O4—C16—C18—C12	179.01 (10)
C1—C2—C4—C5	−0.56 (18)	Co—O11—C11A—O12	172.02 (9)
C2—C4—C5—C6	0.90 (18)	Co—O11—C11A—C12A	−8.37 (19)
C4—C5—C6—C7	−0.62 (18)		

### Hydrogen-bond geometry (Å, º)

**Table:**

*D*—H···*A*	*D*—H	H···*A*	*D*···*A*	*D*—H···*A*
O1*S*—H1*S*···O12	0.84	1.83	2.6671 (14)	174
O2*S*—H2*S*···O1*S*	0.84	1.95	2.7682 (17)	165
O1*W*—H1*W*1···O1^i^	0.80 (1)	1.99 (1)	2.7334 (11)	153 (1)
O1*W*—H1*W*1···O3^i^	0.80 (1)	2.32 (1)	2.9124 (13)	131 (1)
O1*W*—H1*W*2···O2^i^	0.80 (1)	2.18 (2)	2.8071 (11)	136 (1)
O1*W*—H1*W*2···O4^i^	0.80 (1)	2.17 (1)	2.8840 (12)	148 (2)
C9—H9*A*···O1*S*^ii^	0.99	2.52	3.2610 (16)	132
C13—H13*A*···O11^iii^	0.95	2.61	3.5380 (15)	165
C12*A*—H12*B*···O1	0.98	2.38	3.1807 (15)	139
C8—H8*A*···O2*S*^iv^	0.95	2.55	3.4335 (16)	155
C10—H10*A*···O2*S*^ii^	0.99	2.61	3.5119 (17)	151
C12*A*—H12*C*···O2*S*^v^	0.98	2.62	3.5541 (19)	161

Symmetry codes: (i) −*x*+1, −*y*+1, −*z*+1; (ii) *x*−1, *y*, *z*; (iii) −*x*+1, *y*+1/2, −*z*+3/2; (iv) −*x*+1, *y*−1/2, −*z*+3/2; (v) −*x*+2, *y*−1/2, −*z*+3/2.
